# Immune Mediators in Osteoarthritis: Infrapatellar Fat Pad-Infiltrating CD8+ T Cells Are Increased in Osteoarthritic Patients with Higher Clinical Radiographic Grading

**DOI:** 10.1155/2016/9525724

**Published:** 2016-12-14

**Authors:** Jirun Apinun, Panjana Sengprasert, Pongsak Yuktanandana, Srihatach Ngarmukos, Aree Tanavalee, Rangsima Reantragoon

**Affiliations:** ^1^Department of Orthopedics, Faculty of Medicine, Chulalongkorn University, Bangkok, Thailand; ^2^Immunology Division, Department of Microbiology, Faculty of Medicine, Chulalongkorn University, Bangkok, Thailand; ^3^Center of Excellence in Immunology and Immune-Mediated Diseases, Faculty of Medicine, Chulalongkorn University, Bangkok, Thailand

## Abstract

Osteoarthritis is a condition of joint failure characterized by many pathologic changes of joint-surrounding tissues. Many evidences suggest the role of both innate and adaptive immunity that interplay, resulting either in initiation or in progression of osteoarthritis. Adaptive immune cells, in particular T cells, have been demonstrated to play a role in the development of OA in animal models. However, the underlying mechanism is yet unclear. Our aim was to correlate the frequency and phenotype of tissue-infiltrating T cells in the synovial tissue and infrapatellar fat pad with radiographic grading. Our results show that CD8+ T cells are increased in osteoarthritic patients with higher radiographic grading. When peripheral blood CD8+ T cells were examined, we show that CD8+ T cells possess a significantly higher level of activation than its CD4+ T cell counterpart (*P* < 0.0001). Our results suggest a role for CD8+ T cells and recruitment of these activated circulating peripheral blood CD8+ T cells to the knee triggering local inflammation within the knee joint.

## 1. Introduction

Osteoarthritis (OA) is a disease of the joint organ that involves all surrounding tissues, which include articular cartilage, underlining synovium, subchondral bone, menisci, and ligaments, resulting in joint failure. Pathological findings demonstrate breakdown of the cartilage, synovial inflammation, and alteration of periarticular bone structure (marginal osteophyte formation, subchondral bone sclerosis and cyst, bone marrow lesions, and tidemark advancement) [1, 2]. The knee is the second most commonly affected joint for OA [3]. Consequences of knee OA include physical impairment, reduced quality of life, and increased risk for morbidity and mortality as well as economic burden on both affected individuals and society [4, 5].

The knee joint is surrounded by tissues that may serve as initiators of disease progression [1]. The synovial tissue in osteoarthritic patients is characterized by infiltration of immune cells [6–8]. The infrapatellar fat pad has been demonstrated for the presence of immune cells within the tissue and for its ability to secrete cytokines and adipokines, yet providing both a protective role and inflammatory role for the knee [9–12].

Many emerging evidences demonstrate the role of immune responses in the pathogenesis of osteoarthritis [1, 13, 14]. Immune mediators which include both the innate (complement, macrophages, proinflammatory cytokines, and chemokines) and the adaptive (T cells and B cells) compartment come into play in the progression of osteoarthritis [13–17]. Evidence for T cells in the immunopathogenesis of osteoarthritis has been described [16]. However, the underlying mechanisms are still unknown.

In order to investigate how T cells may contribute to the pathogenesis of osteoarthritis, we aimed to phenotypically characterize peripheral blood, synovial tissue, and infrapatellar fat pad T cells and correlate their phenotypes to radiographic grading of knee OA.

## 2. Materials and Methods

### 2.1. Patient Recruitment and Sample Collection

Patients with osteoarthritis undergoing joint replacement (total knee arthroplasty: TKA) at King Chulalongkorn Memorial Hospital were recruited. Peripheral blood was obtained and collected with patient's consent. Synovial tissue and infrapatellar fat pad were obtained as waste materials from the operation. Procedures were performed in accordance with the ethical standards of the responsible committee on human experimentation at the Faculty of Medicine, Chulalongkorn University, Bangkok, Thailand (IRB number 574/57), and with the Helsinki Declaration of 1975, as revised in 2000. Tissue samples obtained were processed immediately.

### 2.2. Radiographic Grading

Preoperative standard plain radiography of all patients was reviewed for radiographic grading according to Kellgren-Lawrence [18] and Ahlback [19] as the following: Kellgren-Lawrence (KL) grading system (grade 0: no radiographic features of OA are present; grade 1: doubtful joint space narrowing (JSN) and possible osteophytic lipping; grade 2: definite osteophytes and possible JSN on anteroposterior weight-bearing radiograph; grade 3: multiple osteophytes, definite JSN, sclerosis, and possible bony deformity; grade 4: large osteophytes, marked JSN, severe sclerosis, and definite bony deformity) and the Ahlback grading system (grade 1: joint space narrowing (joint space < 3 mm); grade 2: joint space obliteration; grade 3: minor bone attrition (0–5 mm); grade 4: moderate bone attrition (5–10 mm); grade 5: severe bone attrition (>10 mm)).

### 2.3. Isolation of Human Peripheral Blood Mononuclear Cell

Peripheral blood mononuclear cells (PBMCs) were isolated from whole blood using the Ficoll-Paque density gradient centrifugation method. Briefly, whole blood was layered on to Ficoll-Paque and centrifuged at 2000 rpm for 20 min without any deceleration force at room temperature. PBMCs were then collected and washed with RPMI 1640 (Gibco, Life Technologies) supplemented with 10% fetal calf serum (FCS) (Gibco, Life Technologies). Cells were cryopreserved in 90% FCS/10% dimethyl sulfoxide (DMSO) (Amresco, USA) until experiments were performed.

### 2.4. Isolation of Mononuclear Cells from Infrapatellar Fat Pad and Synovial Tissue

Tissue samples obtained were washed with phosphate buffer saline (PBS) at pH 7.4 twice prior to sample processing. Tissue samples were then cut into 2-3 mm pieces and digested with phosphate buffer saline (PBS) containing 3 *μ*g/mL collagenase type IV (Worthington), 0.1 *μ*g/mL DNase I (Worthington), and 5% FCS (Gibco, Life Technologies) with shaking at 200 rpm, 37°C for 90 minutes to release cells. Then, supernatant was collected and filtered through a 40 *μ*m filter to remove any undigested residue. Cells were washed with RPMI 1640 (Gibco, Life Technologies) supplemented with 10% FCS (Gibco, Life Technologies) twice. Cells were cryopreserved in 90% FCS/10% DMSO (Amresco, USA) until experiments were performed.

### 2.5. Cell Surface Staining

Cells isolated from peripheral blood and either synovial tissue or infrapatellar fat pad, 10^5^ cells, and cryopreserved cells were labeled with monoclonal antibodies at 4°C for 30 minutes with the following antibodies: anti-CD3-phycoerythrin-cyanine 7 (PE-Cy7) (clone UCHT1), anti-CD4-phycoerythrin-cyanine 5 (PE-Cy5) (clone RPA-T4), anti-CD8-Alexa Fluor 700 (clone SK1), and anti-CD69-phycoerythrin (PE) (clone FN50). All antibodies were purchased from Biolegend, CA, USA. After washing twice the cells with PBS buffer, labeled cells were then fixed and permeabilised with 1% formaldehyde. Cells were acquired on the BD LSRII flow cytometer. Analysis was performed using the FlowJo (Treestar, USA).

### 2.6. Statistical Analysis

All data were analyzed using the Statistical Package for Social Sciences (SPSS 15.0, SPSS Inc., Chicago, IL, USA) and GraphPad InStat version 5.0 software (San Diego, CA, USA). The results were presented as mean ± SD. The demographic parameters of the differences group in OA were compared using one-way analysis of variance (ANOVA). The Mann–Whitney unpaired* t*-test was carried out to determine the differences in the expression of surface marker between groups of OA. Correlation analysis between frequencies of CD4 and CD8 and radiographic severity grading of OA in PBMC, synovial tissue, and infrapatellar fat pad were performed using the one-way analysis of variance (ANOVA). All different data were considered statistical significant at a *P* value of <0.05.

## 3. Results

### 3.1. Demographic Characteristics of Osteoarthritic Subjects

Fifty patients were recruited into the study and classified using the Ahlback classification system and Kellgren-Lawrence (KL) classification system. Patients were classified into Ahlback grades 1 (20%, *N* = 10), 2 (46%, *N* = 23), 3 (28%, *N* = 14), and 4 (6%, *N* = 3) and none with grade 5 severity. However, using the KL classification, over 90% of patients were classified as KL grades 3 (*N* = 24) and 4 (*N* = 25). There were very few or no patients for KL grades 1, 2, and 5. Thus, the Ahlback classification system was used throughout this study.  Table 1 shows the demographic characteristics of patients that were included in the study. We observed here that patients with Ahlback grading 2 had the highest average body mass index (BMI) (Table 1). Either only peripheral blood, synovial tissue, or infrapatellar fat pad; or 2 out of 3 tissues; or all tissues were collected from each patient. Due to the limited amount of tissue, in some samples, isolated lymphocytes were insufficient for further analysis.

### 3.2. Synovial Tissue and Infrapatellar Fat Pad T Cell Infiltration in Knee Osteoarthritic Patients

**Figure 1:**
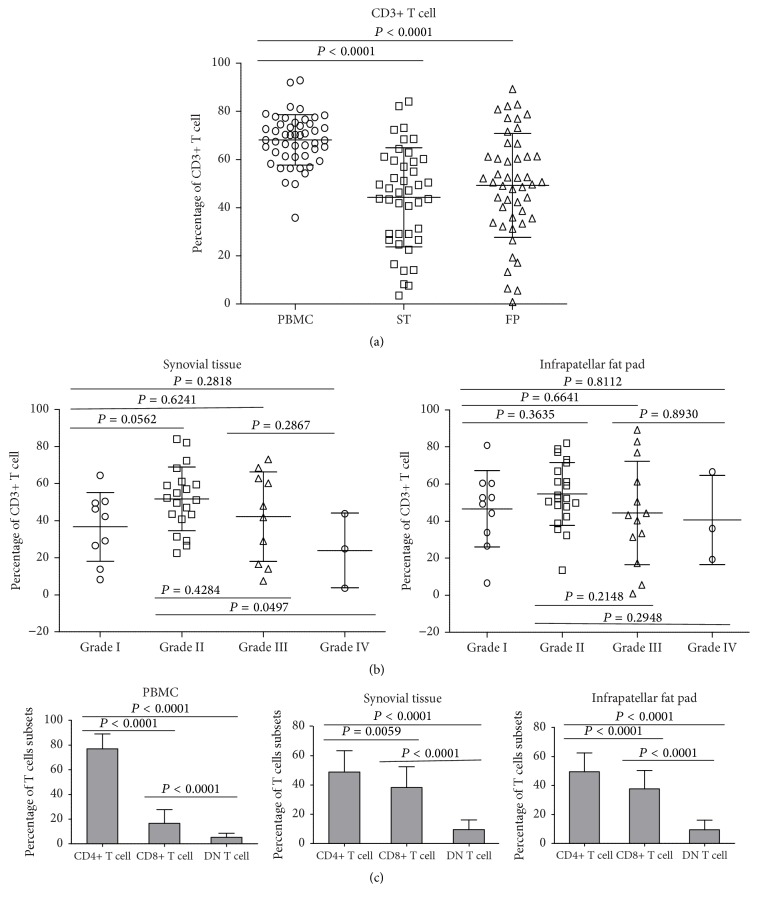
T cell frequency in peripheral blood, synovial tissue, and infrapatellar fat pad of knee osteoarthritis patients. Mononuclear cells were isolated from peripheral blood, synovial tissue, and infrapatellar fat pad. Cells were phenotypically characterized based on CD3+, CD4+, and CD8+ surface expression. (a) Graph comparing CD3+ T cell frequency of total mononuclear cells in peripheral blood (*N* = 48), synovial tissue (*N* = 42), and infrapatellar fat pad (*N* = 47). (b) Graphs showing CD3+ T cell frequency of total mononuclear cells in synovial tissue and infrapatellar fat pad stratified based on Ahlback clinical grading (grades 1–4). Synovial tissue grades I (*N* = 9), II (*N* = 20), III (*N* = 10), and IV (*N* = 3); infrapatellar fat pad grades I (*N* = 10), II (*N* = 21), III (*N* = 13), and IV (*N* = 3). (c) Bar graphs showing composition of different T cell subset (CD4+, CD8+, and DN T cells) frequencies isolated from peripheral blood (PBMC) (*n* = 48), synovial tissue (*N* = 42), and infrapatellar fat pad (*N* = 47). Bars are mean values with error bars.

First, we investigated CD3+ T cells present in peripheral blood, synovial tissue, and infrapatellar fat pad of knee osteoarthritic patients by isolating lymphocytes from these tissues. Flow cytometric analyses revealed the presence of mononuclear cells and CD3+ T cells from these tissues (Figure 1(a)). A substantial proportion of T cell infiltration was found within the synovial tissue (mean = 40.67%) and infrapatellar fat pad (mean = 45.99%), which was observed to be lower than levels in peripheral blood (mean = 69.8%) (*P* < 0.0001) (Figure 1(a)). To determine whether the abundance of T cell infiltration was related to osteoarthritic radiographic grading, patients were stratified using Ahlback's classification and T cell frequency was determined for each group. Our results observed that patient with Ahlback's classification radiographic grade 2 had the highest percentages of T cells in both synovial tissue and infrapatellar fat pad whereas patients with higher grading (grades 3 and 4) had a decline in T cell percentages (Figure 1(b)). Regardless, our findings demonstrate the synovial tissue and infrapatellar fat pad as a site of abundant T cell infiltration in knee osteoarthritis.

### 3.3. Infrapatellar Fat Pad CD8+ T Cell Frequency Is Increased with Higher Radiographic Grading

To further investigate T cell phenotypes that may attribute to the pathogenesis of knee osteoarthritis, we characterized peripheral blood, synovial tissue, and infrapatellar fat pad T cells based on their coreceptor expression into CD4+, CD8+, and DN T cell subsets. In the tissues, the composition of T cell subsets revealed a predominance of CD4+ T cells followed by CD8+ and DN T cells, respectively (Figure 1(c)). However, there was a significantly higher proportion of CD8+ T cell subset in both synovial tissue (mean = 38.75%) and infrapatellar fat pad (mean = 36.86%) when compared to the proportions found in peripheral blood (mean = 17.54%) (*P* < 0.0001) (see Supplementary Figure 1 in Supplementary Material available online at http://dx.doi.org/10.1155/2016/9525724). In contrast, CD4+ T cells were found in a smaller proportion of total T cells in synovial tissue (mean = 48.3%) and infrapatellar fat pad (mean = 49.5%) than in peripheral blood (mean = 75.42%) (*P* < 0.0001) (Supplementary Figure 1). A much smaller increase in frequency of DN T cells was observed in the synovial tissue and infrapatellar fat pad samples when compared to peripheral blood (*P* = 0.0016 and *P* = 0.0001, resp.) (Supplementary Figure 1). These results demonstrate an increase in the proportion of CD8+ T cells within the synovial tissue and infrapatellar fat pad when compared to proportions in the circulating peripheral blood.

**Figure 2:**
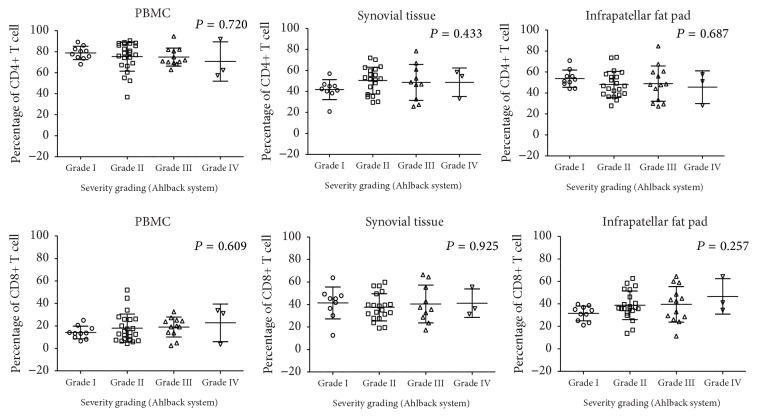
Frequency of CD4^+^ and CD8^+^ T cells from peripheral blood, synovial tissue, and infrapatellar fat pad in patients with higher radiographic grading. Graphs showing frequencies of CD4^+^ and CD8^+^ T cells in peripheral blood (PBMC) (*N* = 48), synovial tissue (ST) (*N* = 42), and infrapatellar fat pad (FP) (*N* = 47) from patients which were clinically graded based on the Ahlback classification system into grades I to IV. Number of dots corresponds to number of the patients. Error bar represents the mean and standard deviation (mean ± SD).

We next correlated the frequency of peripheral blood, synovial tissue, and infrapatellar fat pad CD4+ and CD8+ T cells with the different radiographic grading based on the Ahlback classification system to determine any association between abundance of T cell infiltration and radiographic grading. We observed an increasing trend between peripheral blood CD8+ T cells and infrapatellar fat pad CD8+ T cells with higher radiographic grading whereas peripheral blood CD4+ T cells and infrapatellar fat pad CD4+ T cells exhibited a decreasing trend (Figure 2). In the synovial tissue, we did not observe any trend between both CD8+ and CD4+ T cells with the different grades of radiographic grading (Figure 2).

### 3.4. Peripheral Blood CD8+ T Cells Are Activated in Low Grade Radiographic Severity

**Figure 3:**
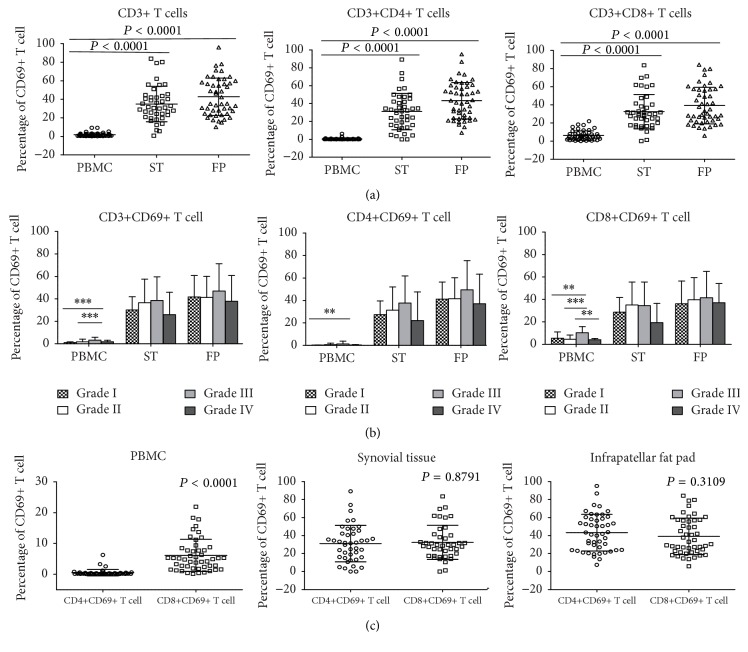
Activation phenotype of T cells isolated from peripheral blood, synovial tissue, and infrapatellar fat pad of knee osteoarthritis patients. Mononuclear cells were isolated from peripheral blood, synovial tissue, and infrapatellar far pad labeled for CD69 surface expression. (a) Graphs showing total T cell and T cell subset (CD4+ and CD8+) frequencies in peripheral blood (PBMC) (*N* = 48), synovial tissue (*N* = 42), and infrapatellar fat pad (*N* = 47). Mean values are shown with error bars. (b) Bar graphs showing the mean frequency of CD69+ total T cells and CD69+ T cell subsets (CD4+ and CD8+) stratified based on the Ahlback classification for radiographic grading. Bars represent mean values with error bars. (c) Comparison of frequency of CD4+CD69+ and CD8+CD69+ T cells in peripheral blood, synovial tissue, and infrapatellar fat pad. Shown are mean values. *P* < 0.05 was considered significant. (^*∗∗∗*^ *p* < 0.0001; ^*∗∗*^ *p* < 0.005; ^*∗*^ *p* < 0.01.)

Next, we compared T cell activation levels of peripheral blood, synovial tissue, and infrapatellar fat pad T cell subsets by determining the frequency of CD69+ T cells. Our results demonstrate a higher level of T cell activation in synovial tissue (*P* < 0.0001) and infrapatellar fat pad (*P* < 0.0001) than in peripheral blood in both total T cell and T cell subset populations. Moreover, we also observed a slightly higher level of T cell activation in infrapatellar fat pads than synovial tissues (Figure 3(a)). We next determined whether higher levels of T cell activation were associated with higher radiographic grading. Similarly as above, we stratified the patients into different Ahlback grading (grades 1–4) and determined the mean level of T cell activation for each grading. Overall, synovial tissue- and infrapatellar fat pad-infiltrating T cells exhibit higher activation levels when compared to T cells isolated from peripheral blood (Figure 3(b)). However, we did not observe any trend between activated T cell (CD69+) frequencies with the different levels of radiographic grading. Surprisingly, we did observe that peripheral blood CD8+ T cells had a significantly higher percentage of activated cells than CD4+ T cells (*P* < 0.0001) (Figure 3(c)) even at lower grades (Figure 3(b)). This was in contrast to peripheral CD4+ T cells, where very few percentages of cells were activated (Figure 3(b)). These results suggest that circulating peripheral blood CD8+ T cells may be in an activated state even prior to recruitment to the osteoarthritic joint.

## 4. Discussion

Our study is among the first few studies to investigate T cells isolated from peripheral blood, synovial tissue, and infrapatellar pad of individuals with knee osteoarthritis. We describe a correlation between infrapatellar fat pad-infiltrated CD8+ T cells with disease severity based on radiographic grading and show that a proportion of peripheral CD8+ T cells are in an activated state, even in patients with low grades of disease severity.

T cells are immune players in the adaptive immune response of many chronic inflammatory diseases. In order to investigate the local inflammation of osteoarthritic joints, we adapted a knee osteoarthritis model in humans and obtained tissues (synovial tissue and infrapatellar fat pad) that surrounded the osteoarthritic joint from patients undergoing TKA. We focused on the relation of T cell frequency and/or the different T cell subset frequency with radiographic grading. Our results reveal a substantial proportion of T cell infiltration within the synovial tissue and infrapatellar fat pad. However, levels were lower than frequencies in peripheral blood as other immune components, such as macrophages, B cells, and mast cells, are composites of this inflammatory cell population [9, 20]. We did not observe any trend in higher Ahlback's radiographic grading with tissue-infiltrating T cell frequency. It is interesting to speculate that patients with radiographic grade 2 had both the highest average BMI values and T cell frequency as obesity (measured by BMI values) is correlated with an increased risk of knee osteoarthritis [21, 22]. In addition, we also observed the decline in BMI values and T cell frequencies in patients with radiographic grades 3 and 4, respectively. It is also alternatively possible that patients with higher grades of severity (grades 3-4) are in a state of more prolonged activation of T cells, thus leading to a loss of expression of CD3 (in particular CD3*ε*) [23]. Earlier studies have reported lower expression of CD3*ζ* chain on synovial T cells in OA patients [24]. On another note, our study was limited on the population size for patients with Ahlback's radiographic grades 3-4. Therefore, we next investigated the different T cell subsets. Many previous studies described infiltration of CD4+ T cells and a Th1/Th2 cytokine profile from synovial tissue lining [6, 7, 20, 25–28]. Our findings confirmed these findings of predominant tissue-infiltrating CD4+ T cells in synovial tissue and infrapatellar fat pad. The proportion of synovial tissue CD4+ : CD8+ T cells have been reported as high as 5 : 1 [8] and were associated with pain score [20]. We report here the same findings of high predominance of synovial tissue CD4+ T cells and, in addition, also in the infrapatellar fat pad. Despite these similar findings, interestingly, we observed a higher proportion of tissue-infiltrating CD8+ T cells in the synovial tissue and infrapatellar fat pad when compared to peripheral blood. Moreover, circulating peripheral blood CD8+ T cells were also found to possess higher levels of activation in our study.

Previously, it has been shown that, in synovial fluid of patients with chronic inflammatory arthritis, Epstein-Barr virus (EBV) peptide-specific CD8+ T cells can be found [29]. Moreover, CD8+ T cells contribute to osteoarthritis progression via cartilage degeneration in an animal model [30]. A higher frequency of human peripheral blood CD8+ T cells was observed in osteoarthritic patients with increasing age which has also been described [31]. Our findings in this study as well as others imply a potential role of CD8+ T cells as immune mediators in osteoarthritis. In addition, genetic factors, which include both HLA classes I and II and a single nucleotide polymorphism (SNP) mapped to an inhibitor of T cell proliferation, were reported to be associated with osteoarthritis [32–39], further underscoring potential roles of T cells in the development of osteoarthritis.

In addition, Attur et al. recently described the use of systemic markers of inflammation as biomarkers of osteoarthritis [40, 41]. This finding supports our observation of relatively higher levels of peripheral blood activated CD8+ T cells when compared to its CD4+ T cell compartment. Both these findings suggest that osteoarthritis may be a disease state of systemic inflammation of the host, despite local clinical symptoms.

Limitations of our study include our selection of patients and a lack of healthy control group. For ethical reasons, only tissues from patients that were undergoing TKA were obtained, thus leading to a selection bias of the patient population as being distributed among KL grades 3-4. In addition, our study lacked synovial tissue and infrapatellar fat pad from age-matched healthy cadaveric individuals.

In conclusion, we demonstrate here that the infrapatellar fat pad may serve as a niche for inflammatory T cells to reside, especially CD8+ T cells, and whether these cells are those of preactivated circulating peripheral blood CD8+ T cells, as described in this study, recruited to the tissue sites or whether the high level of CD8+ T cell activation which predisposes the host to local inflammation in the body when exposed to neoantigens in the knee joint is an aspect that still requires further investigation. We favor the idea that both peripheral blood and tissue-infiltrating CD8+ T cells may play a predominant role in the development of knee osteoarthritis.

## Supplementary Material

Supplementary Figure 1: T cell subsets in peripheral blood, synovial tissue and infrapatellar fat pad in knee osteoarthritis patients.
Isolated mononuclear cells were evaluated for their CD4+ and CD8+ co-receptor cell surface expression. Graphs comparing different T cell frequency of CD4+ T cells, CD8+ T cells and DN T cells in peripheral blood (PBMC) (*n* = 48), synovial tissue (*N* = 42) and infrapatellar fat pad (*N*= 47). Each dot represents one patient. Mean values are shown with error bars.

## Figures and Tables

**Table 1:** Demographic characteristics of patient population.

Demographic data	Ahlback classification system (Mean (SD))				*P* value
	Grade 1 (*n* = 10)	Grade 2 (*n* = 23)	Grade 3 (*n* = 14)	Grade 4 (*n* = 3)	
Age (years)	70.11 (4.73)	67.30 (9.24)	70.36 (7.98)	74.33 (7.51)	0.43
Weight (kilograms)	69.99 (11.63)	68.28 (16.63)	65.39 (10.21)	61.17 (10.42)	0.59
Height (centimeters)	155.67 (6.32)	154.91 (7.93)	153.28 (7.14)	157.00 (14.11)	0.83
Body mass index (kg/m^2^)	25.20 (4.87)	28.39 (6.10)	27.83 (4.02)	24.71 (0.32)	0.36
Body temperature (Celsius)	36.12 (0.72)	36.56 (0.41)	36.40 (0.48)	36.32 (0.16)	0.16
Complete blood count					
Red blood cells (10^6^/ul)	4.59 (0.26)	4.38 (0.49)	4.47 (0.43)	3.88 (0.47)	0.11
Platelet (10^3^/uL)	265.13 (38.00)	262.07 (72.81)	267.50 (51.37)	236.67 (108.74)	0.90
White blood cells (10^3^/uL)	7.54 (2.74)	6.15 (1.60)	7.76 (1.76)	7.03 (3.03)	0.26
Neutrophils (percent)	59.89 (11.40)	59.85 (9.10)	62.45 (7.04)	57.30 (16.19)	0.84
Lymphocytes (percent)	29.25 (8.74)	28.85 (7.30)	27.21 (6.40)	29.33 (12.66)	0.94
