# Supplementary material for: Immune Mediators in Osteoarthritis: Infrapatellar Fat Pad-Infiltrating CD8+ T Cells Are Increased in Osteoarthritic Patients with Higher Clinical Radiographic Grading
Source: Int J Rheumatol. 2016 Dec 14;2016:9525724. doi: 10.1155/2016/9525724 (PMC5192329; doi:10.1155/2016/9525724)
Supplement: Supplementary file 1 — Supplementary Figure 1: T cell subsets in peripheral blood, synovial tissue and infrapatellar fat pad in knee osteoarthritis patients. Isolated mononuclear cells were evaluated for their CD4+ and CD8+ co-receptor cell surface expression. Graphs comparing different T cell frequency of CD4+ T cells, CD8+ T cells and DN T cells in peripheral blood (PBMC) (n = 48), synovial tissue (N = 42) and infrapatellar fat pad (N= 47). Each dot represents one patient. Mean values are shown with error bars. [file 9525724.f1.pdf]

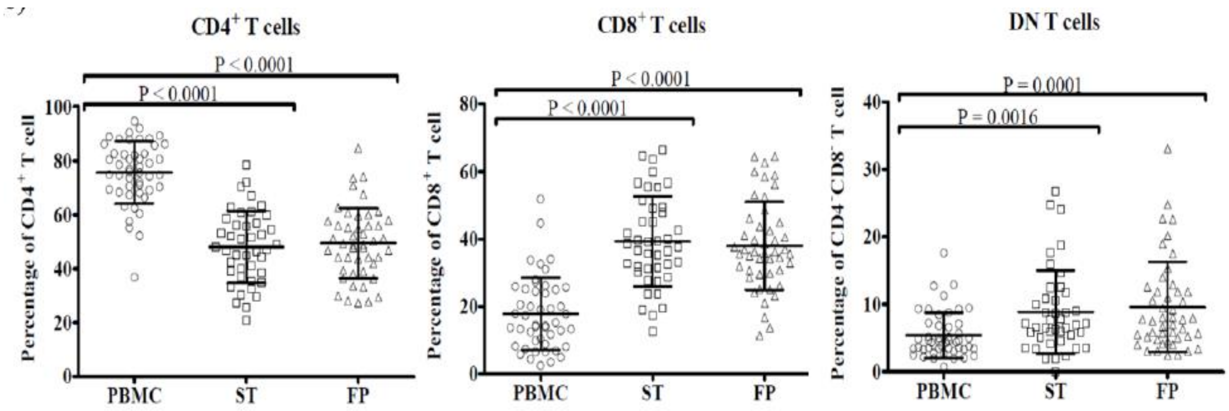

Supplementary figure 1: T cell subsets in peripheral blood, synovial tissue and infrapatellar fat pad in knee osteoarthritis patients.

Isolated mononuclear cells were evaluated for their CD4+ and CD8+ co-receptor cell surface expression. Graphs comparing different T cell frequency of CD4+ T cells, CD8+ T cells and DN T cells in peripheral blood (PBMC) (n=48), synovial tissue (N=42) and infrapatellar fat pad (N=47). Each dot represents one patient. Mean values are shown with error bars.
